# Factors to Consider During Identification and Invitation of Individuals in a Multi-stakeholder Research Partnership

**DOI:** 10.1007/s11606-022-07411-w

**Published:** 2022-02-07

**Authors:** Roses Parker, Eve Tomlinson, Thomas W. Concannon, Elie Akl, Jennifer Petkovic, Vivian A. Welch, Sally Crowe, Marisha Palm, Ana Marusic, Comfort Ekanem, Imad Bou Akl, Michael Saginur, Lorenzo Moja, Tanja Kuchenmüller, Nevilene Slingers, Ligia Teixeira, Laura Dormer, Eddy Lang, Thurayya Arayssi, Regina Greer-Smith, Asma Ben Brahem, Marc Avey, Peter Tugwell

**Affiliations:** 1 Cochrane Pain Palliative and Supportive Care Group, Oxford University Hospitals NHS Foundation Trust, Oxford, UK; 2 Cochrane Gynaecological, Neuro-oncology and Orphan Cancers Group, Royal United Hospitals NHS Foundation Trust, Bath, UK; 3 Population Health Sciences, Bristol Medical School, University of Bristol, Bristol, UK; 4 The RAND Corporation, Santa Monica, USA; 5 Tufts Clinical and Translational Science Institute, Boston, USA; 6 Department of Internal Medicine, American University of Beirut, Beirut, Lebanon; 7 Bruyere Research Institute, Ottawa, Ontario Canada; 8 Crowe Associates Ltd, Thame, UK; 9 Tufts Institute for Clinical Research and Health Policy Studies, Boston, USA; 10 Department of Research in Biomedicine and Health, University of Split School of Medicine, Split, Croatia; 11 Department of Clinical Governance, SERVICOM &E-Health, Ministry of Health, Calabar, Cross River State Nigeria; 12 Montfort Hospital, Montfort Research Institute, Ottawa, Canada; 13 Department of Health Products Policy and Standards, World Health Organization, Geneva, Switzerland; 14 Research for Health Department, Science Division, World Health Organization, Geneva, Switzerland; 15 South African Medical Research Council, Cape Town, South Africa; 16 Centre for Homelessness Impact, Robertson Trust, ESRC, NIHR, London, UK; 17 Future Science Group, Journal of Comparative Effectiveness Research, London, UK; 18 Cumming School of Medicine, University of Calgary, Calgary, Canada; 19 Weill Cornell Medicine, Ar-Rayyan, Qatar; 20 Healthcare Research Associates, LLC/ST.A.R. Initiative, Mountlake Terrace, USA; 21 Instance Nationale de l’Evaluation et de l’Accréditation en Santé (INEAS), Tunis, Tunisia; 22 Public Health Agency of Canada, Ottawa, Canada; 23 Dept of Medicine and School of Epidemiology and Public Health, University of Ottawa, Ottawa, Canada

**Keywords:** Stakeholder engagement, Patient engagement, Patient-centred outcomes research, Research design, International health

## Abstract

**Background:**

Health research teams increasingly partner with stakeholders to produce research that is relevant, accessible, and widely used. Previous work has covered stakeholder group identification.

**Objective:**

We aimed to develop factors for health research teams to consider during identification and invitation of individual representatives in a multi-stakeholder research partnership, with the aim of forming equitable and informed teams.

**Design:**

Consensus development.

**Participants:**

We involved 16 stakeholders from the international Multi-Stakeholder Engagement (MuSE) Consortium, including patients and the public, providers, payers of health services/purchasers, policy makers, programme managers, peer review editors, and principal investigators.

**Approach:**

We engaged stakeholders in factor development and as co-authors of this manuscript. Using a modified Delphi approach, we gathered stakeholder views concerning a preliminary list of 18 factors. Over two feedback rounds, using qualitative and quantitative analysis, we concentrated these into ten factors.

**Key Results:**

We present seven highly desirable factors: ‘expertise or experience’, ‘ability and willingness to represent the stakeholder group’, ‘inclusivity (equity, diversity and intersectionality)’, ‘communication skills’, ‘commitment and time capacity’, ‘financial and non-financial relationships and activities, and conflict of interest’, ‘training support and funding needs’. Additionally, three factors are desirable: ‘influence’, ‘research relevant values’, ‘previous stakeholder engagement’.

**Conclusions:**

We present factors for research teams to consider during identification and invitation of individual representatives in a multi-stakeholder research partnership. Policy makers and guideline developers may benefit from considering the factors in stakeholder identification and invitation. Research funders may consider stipulating consideration of the factors in funding applications. We outline how these factors can be implemented and exemplify how their use has the potential to improve the quality and relevancy of health research.

**Supplementary Information:**

The online version contains supplementary material available at 10.1007/s11606-022-07411-w.

## INTRODUCTION

Patients and the public, providers, payers of health services/purchasers, policy makers, programme managers, peer review editors, and principal investigators are some of the stakeholders involved in decisions aiming to improve individual and public health.^1^ Research leaders increasingly call on the health research enterprise to engage stakeholders in decision-making related to producing and translating evidence into practice.^2–4^

There has been a recent shift in thinking about engagement as exclusively the domain of community-based research, to thinking about it as important to all biomedical sciences, including lab-, clinic-, and hospital-based research. Community-based participatory research sees community stakeholders as equal partners in co-production of research.^5^ Recent stakeholder engagement work draws from community-based participatory research to achieve priorities defined by patient and public communities in defined geographic areas.^5^Community-based participatory research has developed a suite of principles, tools, and processes to help researchers and their nearby communities develop relationships, build trust, and embark on shared research agendas.^6^ The discipline of multi-stakeholder engagement furthers this, calling research teams to engage a wide range of stakeholders.^1^Multi-stakeholder engagement aims to improve the relevance of all forms of health research, policy, and practice by involving a broad range of perspectives, knowledge, and expertise. Broadening the reach of engagement principles has potential to reinforce the shared goal of all bio-medical research becoming patient-centred.

Questions raised by researchers about engaging with stakeholders include the following: what is stakeholder engagement; who should be engaged; how should they be engaged and when; and what difference will it make?.^7^ Multiple stakeholder models have been developed that researchers can use to identify relevant stakeholder groups for involvement in research.^1,8–10^ Overall, these models can be used interchangeably to identify stakeholder groups of interest to a research project, as they include the same stakeholder groups but sometimes with different names to describe them: e.g., ‘patients’ vs ‘patients and consumers’ and ‘clinicians’ vs ‘practitioners’.^11^ Stakeholders can be engaged in research preparation when evidence gaps are identified and questions are prioritised and refined,^12,13^ during research as data are collected and analysed,^7^ following research as dissemination and implementation is planned,^1,9,14,15^ and in evaluation.^16^ By engaging stakeholders to align research evidence with decisions made in seeking, providing, paying for, insuring, and assessing health care, research may become more relevant, better understood, and more widely used in practice.^13,17–20^

Previous research has explored the identification of stakeholder groups to involve in research.^1^ However, there is limited guidance existing to support health research teams in identifying individuals to represent stakeholder groups. Careful and strategic selection of the individuals who represent stakeholders in health research is valued.^21^ This process should involve bi-directional, positive, collaborative, discourse between researchers and stakeholders, aiming to build rapport, trust, and transparent relationships.^16^

We see stakeholder identification as having least two major steps: (1) identification of relevant stakeholder groups for a specific health research topic and consideration of the rationale, extent, roles, and modes of involving stakeholders^11^ , and (2) identification of the individuals to represent each relevant stakeholder group. There is some relevant guidance available in the area of guideline development,^22–24^ but to our knowledge, little exists to guide health research teams in stakeholder identification. This paper seeks to improve current approaches to stakeholder identification by developing factors that can be used to inform the second step.

We propose factors that can be considered by health research teams during stakeholder identification and invitation to ensure a well-balanced research team that is relevant, accountable, and diverse. We use the term ‘research team’ to refer to a group of people working together to reach a common research goal. We define ‘stakeholder’ as ‘an individual or group who is responsible for or affected by health- and healthcare-related decisions that can be informed by research evidence’.^1^ We use the term ‘engagement’ to mean ‘an active partnership between stakeholders and researchers in the research process’.^25^ Our primary audience is health research teams embarking on stakeholder engagement. The many stakeholders that encourage, drive, and support health researchers, such as guideline developers, policy makers, and research funders, are an important secondary audience.

## METHODS

This work has been co-produced by members of the Multi Stakeholder Engagement (MuSE) Consortium, an international network of researchers and stakeholders who share an interest in improving stakeholder engagement in research and guideline development. Author contributions are outlined in Appendix 1.

### Drafting the Factors and Planning Project Methods

Two researchers (ET, RP) developed 18 preliminary factors (Appendix 2), informed by our own experiences with stakeholder mapping and engagement, and originally as part of a stakeholder engagement framework created within Cochrane.^26^ These were refined with MuSE leadership members (PT, TC). We presented the project concept and preliminary factors to the MuSE Consortium in a meeting in February 2021. We invited feedback and formed an executive group of MuSE leadership members (ET, RP, PT, TC, EA, JP, VW) to develop project methods.

### Establishment of the Stakeholder Group

We invited lead representatives from stakeholder groups at the MuSE meeting to contribute to factor development and as co-authors. Sixteen of 22 people accepted, from seven groups: patients and the public (*n* = 3), providers (*n* = 3), payers of health services/purchasers (*n* = 1), policy makers (*n* = 2), programme managers (*n* = 3), peer review editors (*n* = 2), and principal investigators (*n* = 2). Appendix 2 lists group definitions by the MuSE Consortium.

Patient and public representatives involved in this project do not all consider themselves to be academics or researchers. They work with academic partners and stakeholders to ensure the voices of their community and/or patient groups are heard in the decision-making process in health research. Stakeholder representatives (herein referred to as co-authors) are based in Canada, Lebanon, Nigeria, Switzerland, South Africa, Tunisia, the UK, the USA, and Qatar.

### Consensus Phase

We used a modified Delphi process to achieve consensus on the factors. This method allows for consensus based on literature, stakeholder opinions, and expert judgements.^27,28^ We used two stages of feedback from co-authors via email and a final stage involving a teleconference for the executive group to categorise factors lacking consensus.

#### Stage 1

We sought feedback regarding the importance of each of the 18 factors in stakeholder identification, how they could be used by a research team, and examples from co-authors’ experience. We emailed co-authors a draft of the manuscript background and a spreadsheet containing open questions regarding the factors (Appendix 3), asking co-authors to answer the questions, provide comments, and propose new factors. We offered an optional teleconference. We appraised the qualitative responses and looked for consensus with regard to removing or amending factors. We amended the factors and created a document summarising feedback.

#### Stage 2

To determine the importance of the remaining factors in stakeholder selection, we sent co-authors a spreadsheet (Appendix 4), asking them to categorise factors as: ‘highly desirable’, ‘desirable’, or ‘exclude’. We encouraged justifications via a free-text box. To inform this process, we shared the document summarising each factor, created in stage 1.

For each factor, we added votes for ‘highly desirable’, ‘desirable’, and ‘exclude’. We summarised justification given to understand the reasoning behind decisions and contextualise vote counting. We looked for overall agreement and categorised the factors in line with the majority of votes, unless co-author comments conflicted with votes.

#### Stage 3

For factors with no clear consensus, the calculated votes, a summary of comments, and raw comments were presented to the executive group to consider. The executive group held a teleconference to discuss and categorise these factors.

## RESULTS

### Stage 1

We reduced the 18 factors to ten. We merged ‘expertise’ and ‘experience’. We merged ‘Equity’, ‘intersectionality’, and ‘diversity’ under ‘inclusivity’. ‘Motivation’ and ‘capacity’ became ‘commitment and time capacity’. We excluded ‘balance’, ‘power sharing’, and ‘points of view’. See Appendix 5 for the rationale for these changes from co-author feedback. Using feedback, we created a document describing factors, giving ideas of how they could be used, and examples of experiences with the factors (Appendix 6).

### Stage 2

Table 1 presents the final ten factors to consider during identification and invitation of individual representatives in a multi-stakeholder research partnership. We encourage consultation of this table in conjunction with the more detailed table in Appendix 7, which includes specific questions for research teams to consider in this process.

**Table 1:** Factors to Consider During Identification and Invitation of Individuals in a Multi-stakeholder Research Partnership

Factor	Description
Highly desirable	
Ability and willingness to represent stakeholder group	With the caveat that no single person can be expected to represent the views of everyone in a stakeholder group, consider whether the individual stakeholder has the skills required to represent a group and ensure that they are aware of the role they are being asked to fulfil (particularly crucial if the stakeholder identifies as belonging to more than one group).
Commitment and time capacity	Communicate what will be required from the stakeholder, ascertain their commitment, minimise the commitment burden on them, and feed motivation by building rapport.
Communication skills	If a stakeholder can communicate in any way, and they meet other necessary requirements, they should be invited to participate. Adequate budget, time capacity, and resource allocation are important to facilitate good communication by stakeholders and researchers in a multi-stakeholder partnership.
Financial and non-financial relationships and activities, and conflicts of interest	Consider the impact of individuals’ competing relationships and activities on the research and ensure a plan is in place to manage and transparently report these.
Expertise or experience	Seek to create an informed group with a balance of both ‘technical knowledge’ (expertise — which can include e.g. clinical, and methodological) and ‘lived experience’ (experience).
Inclusivity (equity, diversity, and intersectionality)	Consideration of, commitment to, and training in equity, diversity, and intersectionality can ensure stakeholder identification and invitation promotes inclusivity in research.
Training, support, and funding needs	Provide sufficient training, support, and funding to ensure this does not lead to the exclusion of important stakeholder perspectives. Note: if resources are limited and this impacts stakeholder identification and invitation, ensure this is transparently reported.
Desirable	
Influence	Consider the advantages and disadvantages of stakeholders’ level of influence on the research.
Previous stakeholder engagement	Be aware of stakeholders’ previous experience of engaging with research and aim to balance the number of individuals with and without previous experience to include new perspectives and ultimately increase the size and diversity of the pool of available stakeholders.
Research relevant values	Research relevant values such as openness, respect, and integrity may be required, though we recommend personal values not be used to identify stakeholders as this may introduce bias.

Seven factors were categorised as ‘highly desirable’: Ability and willingness to represent stakeholder group; Commitment and time capacity; Communication skills; Financial and non-financial relationships and activities, and conflicts of interest; Expertise or experience; Inclusivity (equity, diversity, and intersectionality); Training, support, and funding needs. Three factors were categorised as ‘desirable’: Influence; Previous stakeholder engagement; Research relevant values.

In Appendix 8, for each factor the numbers of votes for ‘highly desirable’, ‘desirable’, or ‘exclude’ are presented alongside a summary of the qualitative analysis and executive group discussions which provide rationale for the final categorisation.

### Stage 3

Two out of ten factors were split in votes or comments. These were discussed within the executive group teleconference to categorise them as outlined above. Details are provided in Appendix 8.

## DISCUSSION

We present ten factors for research teams to consider when identifying and inviting individuals in a multi-stakeholder research partnership.

### Implementation of the Factors

**Figure 1:**
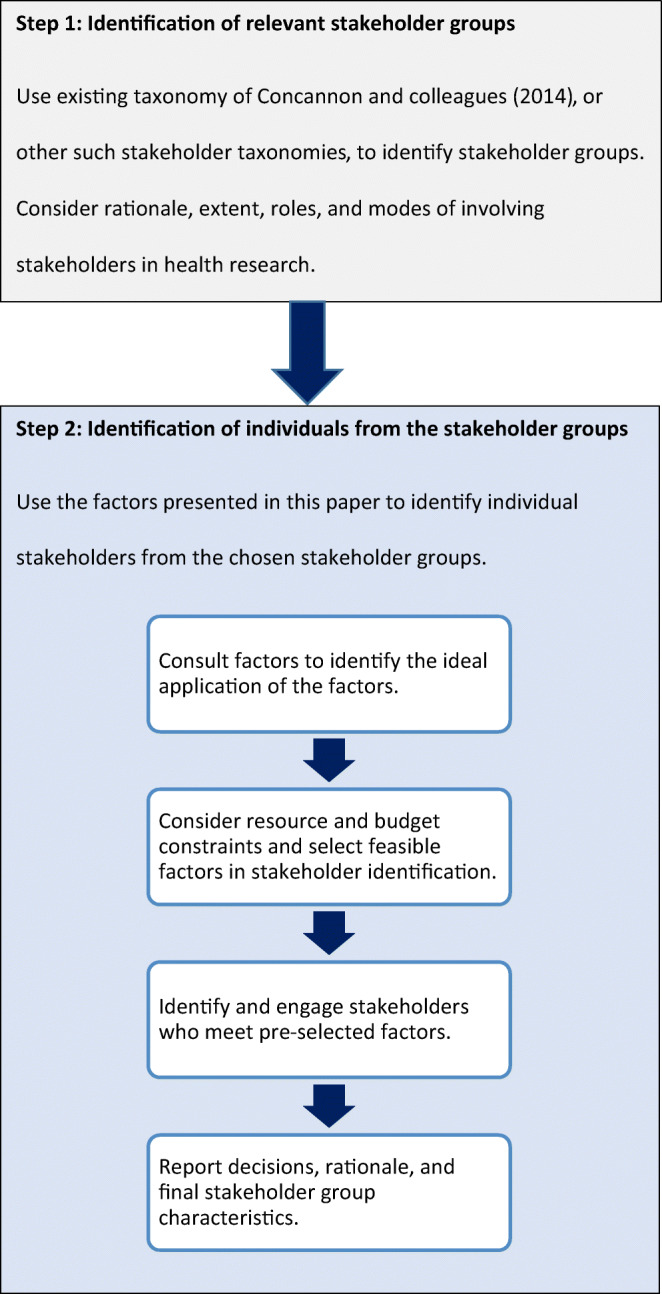
Process of individual stakeholder identification and invitation.

Factors should be considered by health research teams at the beginning of the stakeholder identification process. The extent to which each factor is used by research teams will differ. We have not specified that the factors should be used to determine eligibility. We encourage research teams to use their discretion to make this decision, as their importance and relevance may vary across projects. We suggest the process outlined in Figure 1 for implementation. This involves two phases: (1) identification of stakeholder groups (covered by previous work), and (2) identification of individuals who will represent each stakeholder group (covered by the present factors).

Firstly, begin with the identification of relevant stakeholder groups. This involves careful consideration of the rationale, extent, roles, and modes of involving stakeholders, outlined by Concannon et al..^11^

Secondly, use the factors in this paper to support identification of individuals to represent those groups. First identify the ideal application of these factors. We anticipate ‘highly desirable’ factors will be useful to most research and consideration of these factors will likely have a significant positive influence on the multi-stakeholder partnership. ‘Desirable’ factors may be less applicable and impactful, though we believe considering these will still be beneficial.

Next, consider resource and budgetary constraints to identify a feasible application of the factors. The distinction between ‘highly desirable’ and ‘desirable’ factors may help if resource limitations mean not all factors can feasibly be considered. Thoughtful planning before engagement methods are implemented is important to patients and other stakeholders.^16^

Next, identify and engage stakeholders who meet the pre-selected, feasible, factors. Several methods for stakeholder recruitment exist and resources are available to support researchers.^26,29^

Finally, report decisions made, rationale, and final stakeholder group characteristics. Transparent reporting is important, especially when factors have been used to exclude stakeholders whether by the choice of research team or due to feasibility issues.^13^ We suggest grant applications, protocols, and publications include a section in methods entitled ‘establishment of the stakeholder group’, to specify factors considered in stakeholder identification and how these influenced the research group. Stakeholder engagement reporting templates exist, within which details regarding use of these factors could be included.^13^ Such reporting is already recommended in guideline development, with current guidance asking developers to describe how all contributors to the guideline development were selected, their roles and responsibilities.^23^

Factor assessment and implementation will vary depending on project requirements and research context. Some factors (e.g. previous stakeholder engagement) are easier to assess and take into consideration than others (e.g. research relevant values). A conversation between researchers and stakeholders is often the simplest way to assess stakeholder suitability for engagement. More formal methods include interviews, surveys, and creating a formal assessment process. Qualifications may be ascertained by asking stakeholders for a CV, bio sketch, or references. Appendix 6 provides suggestions regarding assessment and implementation of each factor, as well as examples from real-life experiences.

We are keen to emphasise that stakeholder identification process is two-sided, involving active participation of researchers and stakeholders. Building rapport, trust, and transparent relationships is crucial to engagement success.^16^ Stakeholders will conduct their own assessment of the research team’s suitability to collaborate, perhaps considering many of the same factors, such as whether the research team is able to communicate clearly and provide needed support. Researchers should reflect on their own ability to collaborate with and support stakeholders, and if necessary, undertake training in stakeholder engagement methods. Future research might consider creating a similar tool to that proposed in this paper focusing on research team self-assessment in preparation for stakeholder engagement.

### Strengths and Limitations

We engaged stakeholders representing seven stakeholder groups, from nine countries. We not only involved stakeholders in factor development, we also established an author team of researchers and stakeholders to produce this paper. This team has expertise in health research and lived experience in health care. Despite this, we recognise that our sample is limited by its size and representativeness. We involved 16 individuals, with between 1 and 3 individuals from each stakeholder group. These individuals are the lead representatives for their stakeholder groups within the MuSE Consortium; therefore, we are confident in their ability to fulfil this role effectively. Nonetheless, we acknowledge that these individuals are not representative of all stakeholders.

Due to funding constraints, we were unable to pay public and patient stakeholders, which may have limited their engagement. Due to resource constraints, we did not meet face-to-face with all co-authors to discuss consensus, which may have gathered richer information. However, our online process enabled the inclusion of an international group of co-authors. We were able to obtain detailed feedback effectively and quickly. Furthermore, where there was lack of consensus regarding factor categorisation, the executive group took into consideration co-author feedback, and their own experience and expertise to make decisions on the final categorisation through discussion in a teleconference.

### Implications

This research goes further than previous work focusing on identifying stakeholder groups by providing guidance for the next step of stakeholder selection: the identification and invitation of individuals to represent those stakeholder groups. Use of these factors has potential to help health research teams to form well-balanced, diverse, and informed stakeholder teams. This is likely to improve research quality and applicability.^17–20^ For example, active consideration of the previous experience of stakeholders and an intention to involve a balance of both experienced and novice stakeholders should mean that over time a research area develops a diverse pool of potential stakeholders who possess the skills needed to engage with research. Involvement of these factors is likely to create a research team more accurately reflecting the population it affects. In turn the resultant research will produce more relevant research questions and outcomes, and may be more likely to inform evidence-based decision making.

The factors may also be useful to guideline developers, policy makers, and research funders. Guideline developers and policy makers may benefit from using the factors to select individuals to involve in the guideline or policy development group and to report the process and characteristics of the stakeholder group.^23,30^ Research funders could mandate consideration of these factors for funding decisions. This would incentivise research teams to consider stakeholder involvement early on and protect against this being an afterthought*.*

The usability, acceptability, and usefulness of these factors needs to be assessed in future research. In particular, it is important for the applicability of this work to be explored with larger sample sizes and from the perspective of potentially impacted, under-represented stakeholders, who may be from historically excluded groups. We recognise there are barriers to recruiting people from under-represented stakeholder groups and contacting people who are not connected to the health system. Further research is necessary to ensure that using these factors does not lead to the exclusion of important perspectives embedded within stakeholder groups, such as from individuals in hard-to-reach populations. Application of these factors requires resource and we suggest research teams should be adequately funded to be able to effectively undertake stakeholder identification, promote inclusion, and seek a balance of views.

Overall, this work contributes guidance to support health research teams in identifying individual stakeholders to involve in their work. We believe the process of being involved in research will be more rewarding and research quality will be increased if researchers carefully consider stakeholder identification and invitation, taking time to thoughtfully match stakeholders to the needs of the project, whilst ensuring stakeholders’ needs are also met.

## Supplementary Information

ESM 1 (DOCX 160 kb)
