# Supplementary material for: Factors to Consider During Identification and Invitation of Individuals in a Multi-stakeholder Research Partnership
Source: J Gen Intern Med. 2022 Feb 7;37(16):4047–53. doi: 10.1007/s11606-022-07411-w (PMC9708980; doi:10.1007/s11606-022-07411-w)
Supplement: Supplementary file 1 — (DOCX 160 kb) [file 11606_2022_7411_MOESM1_ESM.docx]

# Appendices

## Appendix 1 - Author contributions

| **Author initials** | **Contributions** |
| --- | --- |
| RP, ET, TC, and PT (members of the ‘executive group’) | Initial conceptualisation of the project including the formulation of the overarching research aims and the development of the preliminary factors. |
| RP, ET, TC, PT, JP, VW and EA (full ‘executive group’) | Refined the factors and developed project methods. |
| TC, PT, JP, VW and EA (members of the ‘executive group’) | Attended regular meetings providing guidance to ET and RP throughout the running of the project |
| RP and ET (lead authors and members of the ‘executive group’) | Created data collection materials, collected data, verified the underlying data, analysed data, with TC wrote the original draft manuscript and revised the manuscript |
| MP, SC, RGS, CE, IBA, MS, LM, ABB, MA, NS, LT, LD, AM, EL, TK, TA (16 stakeholders) | Contributed data to stage 1 and stage 2 of the factor development. Commented on the manuscript. |
| All authors | In line with ICMJE criteria for authorship, all authors contributed to the conception of the work or the acquisition, analysis, or interpretation of data. All authors contributed to either drafting or revising the work. All authors gave final approval of the version to be published and agreed to be accountable for all aspects of the work. |

## Appendix 2 – Definitions of the stakeholder groups involved in this project, produced by the Multi-Stakeholder Engagement Consortium

| **Stakeholder groups** | **Definition** |
| --- | --- |
| Patients and the public | A patient is an individual who has experience with the condition/disease of interest. There public are the general population within a defined geographic area, excluding patients, caregivers and health professionals, living or working with the condition of interest |
| Providers | Persons - and their professional associations - who provide health care in a professional capacity (i.e., front line health workers - clinicians, pharmacists, a person who is qualified and allowed by regulatory bodies to provide a health care service) |
| Payers of health services/ purchasers | Individuals, organizations and entities that pay for health services (e.g., provincial health plan, purchasers of drugs and devices, Kaiser (HMO), public health system, private insurers) |
| Policy makers | Individuals, organizations and entities that craft public or private policy (on health) at any level of government (e.g., politicians, national, provincial, state, or local, scientific advisors) |
| Program managers | Entities that deliver health care or programs to patients (e.g., manager of a health care program (e.g. palliative care)) |
| Peer review editors | Individuals who manage peer review or edit peer reviewed research |
| Principal investigators | Individuals, organizations, and associations that conduct or advocate health research (i.e., researchers and all members of the research team) |

## Appendix 3 - Spreadsheet containing the original 18 factors and open questions about each factor, used in stage one

| **Instructions to co-authors:**  To develop the list of stakeholder identification factors, we list all 18 factors below for your consideration and comment.  Please consider each of the stakeholder identification factors and respond to the prompt questions beneath each one. Please do not feel restrained by the prompt questions, we are keen to hear your feedback even if not in answer to a question! Please give as much detail as possible. For each factor we welcome examples, or full case studies, from your work to show where the factor has been relevant (or not). In addition please consider whether there are factors missing from the list. You will have an opportunity to state additional factors and answer general questions about the list as a whole at the end of the document.  Thanks again for your input in this project! | | | |
| --- | --- | --- | --- |
|  |  |  | |
| Name: |  |  | |
| Stakeholder group (P) |  |  | |
|  |  |  | |
| Factor | Probe question | Comments |  |
| Expertise | 1.       Is it important for a research team to consider whether a stakeholder has expertise in the topic area and/or activity the research team is working on? Please give your rationale for your answer. |  |  |
|  | 2.       How could a research team assess a stakeholder’s expertise? |  |  |
|  | 3.       Please add other comments/ examples from your experience related to this factor here: |  |  |
| Experience | 1.       Is it important for a research team to consider whether a stakeholder has experience in the topic area and/or activity they are working on? Please give your rationale for your answer. |  |  |
|  | 2.       How could a research team assess a stakeholder’s experience? |  |  |
|  | 3.       Please add other comments/ examples from your experience related to this factor here: |  |  |
| Influence | 1.       Is it important for a research team to consider whether a stakeholder has influence in the topic area and/or activity they are working on? Could this lead to negative selection bias? Please give your rationale for your answer. |  |  |
|  | 2.       How could a research team determine a stakeholder’s influence? |  |  |
|  | 3.       Please add other comments/ examples from your experience related to this factor here: |  |  |
| Values | 1.       Is it important for a research team to consider a stakeholder’s values? Please give your rationale for your answer. |  |  |
|  | 2.       How could a research team assess stakeholder values? |  |  |
|  | 3.       Is it important for the stakeholder’s values to match those of the research team? |  |  |
|  | 4.       Would it be useful or possible for this paper to posit a list of universal values that all research teams should adopt and look for in stakeholders, or is it more appropriate for each research team to have its own values? |  |  |
|  | 5.       Please add other comments/ examples from your experience related to this factor here: |  |  |
| Point of view | 1.       Is it important for a research team to consider a stakeholders point of view when identifying stakeholders? Please give your rationale for your answer. |  |  |
|  | 2.       How could a research team assess stakeholder points of view? |  |  |
|  | 3.       Do you think diversity in points of view is important in a research team? |  |  |
|  | 4.       Should points of view be balanced in a researcher-stakeholder group? |  |  |
|  | 5.       What is the difference between values and point of view? |  |  |
|  | 6.       Please add other comments/ examples from your experience related to this factor here: |  |  |
| Previous engagement | 1.       Is it important for a research team to consider previous engagement with a stakeholder? i.e. to consider whether the impact of previous work could be maximised or different work undertaken with the same stakeholder, or whether it is more appropriate to seek input from new stakeholders. Please give your rationale for your answer. |  |  |
|  | 2.       How can we ensure we carefully address this item to avoid known examples where researchers/ guideline developers select stakeholders they have worked with before (‘the usual suspects’) because they know they will agree with them? |  |  |
|  | 3.       Please add other comments/ examples from your experience related to this factor here: |  |  |
| Communication skills | 1.       Is it important for a research team to consider a stakeholders communication skills? i.e. ability to communicate clearly and the practicalities of communication such as geographical location, time zone, and whether specialist equipment will be needed for those with different modes of communication (e.g. sign language). Please give your rationale for your answer. |  |  |
|  | 2.       How could a research team assess stakeholder communication? |  |  |
|  | 3.       Is it important that people with poor communication skills are not excluded from the team, but the researcher is skilled to adapt to ensure inclusivity and facilitate the discussion? |  |  |
|  | 4.       Please add other comments/ examples from your experience related to this factor here: |  |  |
| Power sharing | 1.       Is it important for a research team to consider a stakeholders ability to share power with others? Please give your rationale for your answer. |  |  |
|  | 2.       How could a research team assess whether people have the ability and willingness to share power? |  |  |
|  | 3.       Is this factor more a responsibility of the research team to have the skills in dealing with power imbalances? |  |  |
|  | 4.       Please add other comments/ examples from your experience related to this factor here: |  |  |
| Capacity | 1.       Is it important for a research team to consider a stakeholders capacity (e.g. time and resources) to engage? Please give your rationale for your answer. |  |  |
|  | 2.       How could a research team assess stakeholder capacity? |  |  |
|  | 3.       How can researchers encourage those who may not have capacity? |  |  |
|  | 4.       Please add other comments/ examples from your experience related to this factor here: |  |  |
| Motivation | 1.       Is it important for a research team to consider a stakeholders motivation to engage with them and to collaborate with others? Please give your rationale for your answer. |  |  |
|  | 2.       How could a research team assess stakeholder motivation? |  |  |
|  | 3.       How can researchers inspire motivation in potential stakeholders? |  |  |
|  | 4.       Please add other comments/ examples from your experience related to this factor here: |  |  |
| Training | 1.       Is it important for a research team to consider the amount of training and support the stakeholder will require, and whether they have the capacity to provide it? Please give your rationale for your answer. |  |  |
|  | 2.       How could a research team assess stakeholder training needs? |  |  |
|  | 3.       Do you think it is important for the research team to assess the training they themselves may need to work effectively with the stakeholder? |  |  |
|  | 4.       Please add other comments/ examples from your experience related to this factor here: |  |  |
| Equity | 1.       Is it important for a research team to consider the impact on equity when identifying stakeholders to involve in research? Please give your rationale for your answer. |  |  |
|  | 2.       How could a research team consider this? Do you think it might be useful to consider the PROGRESS-Plus characteristics (see: https://methods.cochrane.org/equity/projects/evidence-equity/progress-plus), developed by the Equity Methods Group, when identifying a stakeholder group? |  |  |
|  | 3.       Please add other comments/ examples from your experience related to this factor here: |  |  |
| Intersectionality | 1.      Is it important for research teams to take an intersectional approach when identifying stakeholders to involve in research, by acknowledging that institutionalised forms of oppression (i.e. racism, sexism, and classism) exist and considering the interaction between social identity and social structures? Please give your rationale for your answer. |  |  |
|  | Note: this factor has been developed using resources produced by the Knowledge Translation Program (see: https://knowledgetranslation.net/portfolios/intersectionality-and-kt/). As defined by the Knowledge Translation Program ‘Intersectionality is a way of looking at the world that recognizes that people’s experiences are shaped by a combination of social factors, including their gender, racialization, age, among others. These experiences occur within and interact with connected systems and structures of power, such as sexism and racism.’ |  |  |
|  | 2.      Is it important for research teams to consider how their own biases (i.e. preconceived judgements for or against a particular individual or group) or their organisation’s policies or practices could create advantages or disadvantages to participating based on someone’s intersecting categories (i.e. including age, gender, identity, sex)? |  |  |
|  | 3.       Please add other comments/ examples from your experience related to this factor here: |  |  |
| Diversity | 1.       Is it important for a research team to consider diversity in the stakeholder group? Please give your rationale for your answer. |  |  |
|  | 2.       How can researchers ensure diversity in a stakeholder group? |  |  |
|  | 3.       Please add other comments/ examples from your experience related to this factor here: |  |  |
| Balance | 1.       Is it important for a research team to seek a balanced stakeholder group? Please give your rationale for your answer. |  |  |
|  | 2.       How can researchers ensure balance? |  |  |
|  | 3.       Balance may not always be achievable or feasible, in this case, can you suggest any strategies to mitigate an unbalanced group? |  |  |
|  | 4.       Please add other comments/ examples from your experience related to this factor here: |  |  |
| Representation | 1.       Is it important for a research team to consider whether stakeholders have the ability to represent their stakeholder group? Please give your rationale for your answer. |  |  |
|  | 2.       How can researchers assess if stakeholders have the ability to think beyond their personal experience to represent their stakeholder group? |  |  |
|  | 3.       Please add other comments/ examples from your experience related to this factor here: |  |  |
| Funding | 1.       Is it important for a research team to consider whether it is appropriate for a stakeholder to receive funding for participation whilst identifying stakeholders? Please give your rationale for your answer. |  |  |
|  | 2.       How could researchers assess this? What level of input from stakeholders would warrant funding? |  |  |
|  | 3.       Please add other comments/ examples from your experience related to this factor here: |  |  |
| Conflict of interest | 1.       Is it important for a research team to consider conflict of interest when identifying stakeholders? Please give your rationale for your answer. |  |  |
|  | 2.       Do individuals representing a specific stakeholder group have an inherent conflict of interest related to that representation? Or should the interests of the stakeholder group that the individual is representing not be considered a conflict of interest, as this is the point of their inclusion in the project? |  |  |
|  | 3.       Should the interests of the individual representing the stakeholder group, however, be carefully considered for potential conflicts and managed accordingly? These conflicts could be: |  |  |
|  | i.      At the individual level e.g. financial (direct financial benefit/ benefit through professional status), intellectual or personal. |  |  |
|  | ii.      Through institutional affiliation e.g. financial (direct financial benefit to institution or benefit through increasing services provided by institution – note: financial conflicts are often defined differently between countries) or cultural. |  |  |
|  | 4.       How can potential/ perceived conflicts of interest be assessed when identifying stakeholders? |  |  |
|  | 5.       Can research teams and should research teams assess whether stakeholders have an open mind? |  |  |
|  | 6.       Please add other comments/ examples from your experience related to this factor here: |  |  |
| General questions about the overall list | 1.       Are there any additional factors important for consideration in stakeholder identification missing from this list? Please state them along with your reasoning. |  |  |
|  | 2.       Do you think any of the factors above are not necessary to consider in stakeholder identification? |  |  |
|  | 3.       The above factors are mainly worded to consider the traits of the stakeholder in stakeholder identification, however some reflect also on the research team (e.g. assessing communication skills/ ability to share power in the stakeholder but the researcher also needs skills in facilitating communication and power sharing). Do you think it necessary to have distinct factors for the research team to consider in self-assessment? |  |  |
|  | 4.       What are potential barriers to the use of these stakeholder identification factors? How can we encourage researchers to use it in stakeholder identification? |  |  |
|  | 5.       Please add other comments here: |  |  |

## Appendix 4 - Spreadsheet with ten factors for categorisation in stage two

| **Instructions to co-authors:**  Please find below the amended set of stakeholder selection factors. The factor list has been amended following your feedback. For an explanation of each factor, please read the word document we emailed to you alongside this excel sheet. In this exercise, we ask you to please select a 'grouping' for each of the factors using the dropdown menu next to each factor. The options are highly desirable, desirable, or exclude. In row D, there is space for you to provide reasoning for your decision. We will analyse your responses to group the factors into these categories, for reporting in the manuscript. Thanks again for your input in this project! | | | |
| --- | --- | --- | --- |
|  |  |  | |
| Name: |  |  | |
| Stakeholder group (P) you are representing: |  |  | |
|  |  |  |  |
| Factors for consideration by research teams when selecting stakeholders | Grouping | Comments |  |
| Expertise or experience |  |  |  |
| Influence |  |  |  |
| Research relevant values |  |  |  |
| Previous stakeholder engagement |  |  |  |
| Ability and willingness to represent stakeholder group |  |  |  |
| Inclusivity (equity, diversity and intersectionality) |  |  |  |
| Communication skills |  |  |  |
| Commitment and time capacity |  |  |  |
| Conflict of interest |  |  |  |
| Training, support, and funding needs |  |  |  |

## Appendix 5 – Results from analysis in stage 1, showing the process behind reducing the factors from 18 to 10

| **Original 18 factors** | **10 factors following stage 1** | **Reasoning for changing, merging, or excluding factors following qualitative analysis** |
| --- | --- | --- |
| Expertise | Expertise or experience | Co-authors noted that including some stakeholders with expertise or experience in a research team is useful. However, expertise might be more relevant for certain types of stakeholders (e.g., principal investigators, policy makers) while experience might be more relevant to other types (e.g., patients, public). Therefore, factors were merged, making it ‘expertise **or** experience’. |
| Experience |  |  |
| Influence | Influence | Factor remained the same. |
| Values | Research relevant values | Co-authors noted that ‘values’ was too broad as it could encompass personal values and research relevant values. Co-authors felt personal values should not be considered in stakeholder selection as it may be constraining, introduce bias, and be difficult to assess. Research relevant values were judged a worthwhile consideration at this stage. Therefore, the title was changed to ‘Research relevant values’. |
| Previous engagement | Previous stakeholder engagement | Factor remained the same. |
| Representation | Ability and willingness to represent the stakeholder group | Co-authors noted being unclear on the definition of ‘representation’ in this context. Title changed to make clear that this factor refers to a stakeholder’s ability and willingness to represent the stakeholder group. |
| Equity | Inclusivity (equity, diversity, and intersectionality) | Co-authors noted that these factors are interlinking and can be combined. Factors were merged under the title ‘Inclusivity (equity, diversity, and intersectionality). |
| Intersectionality |  |  |
| Diversity |  |  |
| Communication skills | Communication skills | Factor remained the same. |
| Motivation | Commitment and time capacity | Commitment was suggested by a co-author. Motivation is covered within this factor. Capacity was renamed ‘time capacity’ to be more specific. Motivation and capacity were merged to make ‘Commitment and time capacity’. |
| Capacity |  |  |
| Conflict of interest | Financial and non-financial relationships and activities, and conflicts of interest. | Title changed to reflect recent changes in the definition by the International Committee of Medical Journal Editors (ICMJE). |
| Training | Training, support, and funding needs | Training and funding were combined as both factors reflect something research teams need to provide in response to stakeholder requirements. Support was added to cover resources other than training and funding. |
| Funding |  |  |
| Balance | Excluded | Co-authors felt balance of perspectives is important in a researcher-stakeholder team and this will be facilitated using all these factors, making this specific factor redundant. |
| Power Sharing | Excluded | Co-authors felt facilitating power sharing is related more to the skill of the researcher than something that should be considered about a stakeholder. |
| Points of view | Excluded | Assessing and defining points of view was perceived too difficult in stakeholder selection. A stakeholder’s point of view is changeable over time and some co-authors felt this was over-management. |

## Appendix 6 – Summary document created with feedback from stage 1

Note: Descriptions of items have changed since this document was created following feedback and discussions in stage 2 of the project. Find the full final descriptions of the factors and important considerations for researchers utilising the factors in Appendix 7 and find a summary of the factors in the Table in the manuscript.

Expertise or experience

Research teams should involve stakeholders with relevant expertise or experience. We recognise the importance of including individuals with relevant technical knowledge (e.g., clinical, and methodological expertise) or lived experience in a multi-stakeholder engagement plan. Some stakeholders may be more likely to have lived experience than technical expertise (e.g., patients and the public); others may be more likely to have technical expertise than lived experience (e.g., principal investigators and policy makers).

*How can this factor be assessed/implemented?*

We recommend the research team establish what expertise or experience is required in the first instance, before assessing this in stakeholders. The following are potential methods to assess stakeholder expertise or experience:

- Ask for CV, or bio sketch,
- Ask for a case study / letter of intent describing experience
- Ask for references,
- Ask for examples of expertise in the required field,
- Have an informal conversation or formal interview,
- Conduct a web search of the individual,
- Ask stakeholders to report the primary stakeholder group they would like to be identified in representing and to describe their technical knowledge or lived experience with the research topic.

*Quotes from co-authors describing examples of each criterion*

I have had public stakeholders complete brief, appropriately aimed applications, and also had informal interviews to assess interest, experience, and expertise.

I have asked for a relevant biography or pen portrait to show their relevance and experience in the field of interest.

We have asked the person to write a case study of their experience and this has helped check their actual ability as well as reasoning and communication.

When developing a stakeholder list for a new NICE guideline, we started by co-creating the list together (we brought the homelessness expertise) but we then involved other key people and a wider group to ensure we were looking at 'expertise' from multiple lenses (rather than clinical or social sciences expertise).

Here are some examples in regard to what I have done to select individuals with experience:

1) Selecting chief of divisions,

2) selecting nurses who work predominantly with patients in the field of study,

3) reaching out to physicians who work in high-capacity hospitals and clinics as potential candidates.

Influence

Research teams should consider whether a stakeholder has influence within the research area, the type of influence and how it may affect the work. Influence may positively affect your project through improved dissemination and uptake. It may also negatively influence your project through pre-existing loyalties and pre-conceived notions. Selecting those with influence may also lead to inequity. It is important to be cognizant of variation in influence and to build safeguards against power imbalances while not casting influence away.

*How can this factor be assessed/implemented?*

Stakeholder influence can be assessed using:

- Membership to academic, social, or health networks, institutions, bodies, or organisations,
- Social media profiles (e.g., LinkedIn, Twitter, Facebook, Instagram),
- Publication profile, citation index,
- Questions to them about contacts/ relationships with relevant groups/ organisations/ people,
- Bio sketch, 360-degree references, CV,
- Attendance at relevant conferences.

*Quotes from co-authors describing examples of each criterion*

In a nurse-led study on early discharge of new-borns and mothers … engaging the most senior & experienced clinicians in the speciality groups got them on board to use their influence to overcome potential barriers to rolling out the program (which might have stopped less influential individuals).

I have found that information about links to other individuals, related groups, or community organizations can be very helpful in understanding what networks the person carries with them. I have found many people who are chosen for their ability to influence but who then can’t understand the issues and hence become poor representatives.

Research relevant values

We differentiate between research relevant values and personal values.

A research project may require stakeholders to accept a universal set of research relevant values, or a set of research relevant values adopted by the research team. Examples include openness, respect, equipoise, and integrity. It is important for the research team to be clear about values for research and explore whether the stakeholder representative shares these.

At the same time, research teams should not consider personal values when selecting stakeholders as this could bias the selection of stakeholders to be more like the research team, and lead to groupthink. This would potentially result in stakeholders being more deferential to the perceived desires of the research team, which may undermine stakeholder function as a representative.

*How can this factor be assessed/implemented?*

It is hard to assess values effectively. Researchers could make individuals aware of the research group’s values (e.g., as part of their code of conduct) and ask them to sign an attestation of awareness of the values before joining the project. Other options include:

- Speaking with the stakeholder informally or via interview,
- Examining what attracted the stakeholder to the group,
- Asking the stakeholder via survey ‘how much do you value ___ in a team?’,
- Examine their organisation’s mission statement,
- Social media postings.

*Quotes from co-authors describing examples of each criterion*

We had values for the James Lind Alliance of inclusivity, transparency etc with examples after each of what this meant in reality.

…in my practice I work within the stated values; values for quality, confidentiality, consistency, time etc.

The process of agreeing values can be a very useful one, especially before digging into topics where there are different perspectives; it reminds the team that they hold similar goals for the work and are just talking about different ways of getting to the same end. I find it useful to constantly remind people of these shared values to keep alignment and on track- it also helps stimulate questions and thinking in the group.

If values for responsible research are important here, then those outlined in the European Code of Conduct for Research Integrity are relevant (Reliability, Honesty, Respect, Accountability) or those from the US National Academies of Sciences, Engineering, and Medicine – Fostering Integrity in Research (Accountability, Honesty, Objectivity, Openness, Stewardship, Fairness), see: <https://pubmed.ncbi.nlm.nih.gov/33502638/>

Previous stakeholder engagement

It is important for a research team to consider whether to involve stakeholders they have worked with previously or new stakeholders in their research project. There are advantages and disadvantages for either option. It can take time to train both the research team and stakeholders to work effectively together. Working with the same individuals over time can be a positive sign of a healthy, productive working relationship.

However, we are keen to encourage research teams to involve new stakeholders in new projects. Different individuals might contribute a lot to the research project through different expertise and/ or experience and fresh perspectives. Involving new stakeholders will also increase the size and diversity of the pool of experienced stakeholders to engage in research.

*How can this factor be assessed/implemented?*

When considering the selection of stakeholders with whom they have had vast previous engagement, research teams need to assess the risk of acquiescence: *‘If you always do what you've always done, you'll always get what you already got’*. If researchers are struggling to select new stakeholders, they could consider ensuring a rotation in their expert advisors, establishing a diversity/early career researcher policy, or looking for stakeholders in different but related fields.

*Quotes from co-authors describing examples of each criterion*

I have seen members of the public who have worked with health researchers for years, providing strong and useful feedback that challenged thinking and led to better research.

Ability and willingness to represent the stakeholder group

Stakeholders need to have the skills to take on the role of stakeholder representative effectively, including the ability to look beyond their personal situation, imagination, and empathy. A single individual is not expected to represent all perspectives of a certain stakeholder group.

On the other hand, an individual may belong to more than one stakeholder group. For example, a practitioner may also have a family member with a condition meaning they can inform more than one perspective. The research team should recruit such an individual to represent only one stakeholder group and should clarify to that individual which stakeholder group they are being asked to represent whilst being aware of the other perspectives that individual holds.

The above points must be made clear to the stakeholder from the beginning.

*How can this factor be assessed/implemented?*

Prior to stakeholder engagement, it is difficult to assess the extent to which an individual can think beyond their personal circumstances to represent others. Assessment can be conducted by considering an individual’s CV and information on previous experience, a pre-engagement survey, and an initial meeting formal or informal. It may also be useful to recruit more than one individual representing each group.

*Quotes from co-authors describing examples of each criterion*

I'm Jewish. Sometimes people have expected me to act or think not as I do, because of that. I don't think a researcher should judge how well I can represent Jews based on their concept of how Jews are.

In the interest of parsimony, I often recruit only one individual to represent each of the relevant professional Ps (those with salary and institutional backing) and more than one to represent patients, consumers, and other members of the public.

Inclusivity (equity, diversity, and intersectionality)

Research teams should be committed to actively and openly supporting and promoting inclusivity by considering **equity, diversity and intersectionality** when identifying stakeholders to involve in a research project.

Research teams should adopt an ‘equity lens’ throughout stakeholder identification and aim to promote health equity, defined as absence of avoidable and unfair differences in health(1). One way to promote health equity is to include ‘vulnerable’ stakeholders. When identifying stakeholders, it is important for research teams to consider the characteristics and demographics of the population that the research aims to serve and ensure that stakeholder selection reflects this diversity. This increases generalizability and usefulness of results to those who need them.

The research team should also consider intersectionality, i.e., the intersections among different social hierarchies, such as class and gender, and their cumulative impacts on health inequities Individuals may be affected by discriminatory social structures in more than one way (2). Research teams should consider how their own biases (for example, preconceived judgements for or against a particular individual or group), or their organisation’s policies or practices, could disadvantage individuals’ intersecting categories (i.e., including age, gender, identity, sex).

We acknowledge that these concepts may have different understandings and interpretations in different countries and communities, therefore this factor may be culturally sensitive. The research team may benefit from training in these issues to be able to effectively address them.

*How can this factor be assessed/ implemented?*

Research teams should consider inclusivity early on during the process and make it a priority.

Research teams might benefit from considering [PROGRESS-Plus characteristics](https://methods.cochrane.org/equity/projects/evidence-equity/progress-plus) – an acronym developed by the Cochrane and Campbell Equity Methods Group and used to identify characteristics that stratify health opportunities and outcomes. Certain characteristics from the PROGRESS-Plus list may be prioritised depending on the research project and scope.

Ideas for improving diversity include:

- Broadening networks to include diverse groups,
- Taking time to engage with different groups,
- Selecting stakeholders based on pre-determined characteristics,
- Completing thorough stakeholder identification mapping exercise,
- Including multiple stakeholder groups.

[Materials produced by the Knowledge Translation Program](https://knowledgetranslation.net/portfolios/intersectionality-and-kt/) may be useful to research teams wanting to learn more about intersectionality, how to assess their own biases and how to consider this when selecting stakeholders.

*Quotes from co-authors describing examples of each criterion*

We have set targets for stakeholders before- both looking at sex, sexual orientation, and race. If you are not pro-active about this, it is easy to find yourself struggling with equity. The type of equity you want to achieve could also be expanded for specific circumstances- for example nothing for us without us.

A racial and social justice perspective on equity says that overweighting and over-representation of historically excluded groups may be necessary to rectify structural injustice

In my limited experience of extended discussions that considered intersectionality (in ‘cultural safety training’ that focused on First Nations https://www.sanyas.ca/), I found that listed characteristics (e.g., white settler female ... ) were fairly uninformative in terms of what I took away from the discussion ... people's narratives were a much richer source of information, but then I don't know how to systematically and rigorously approach meaningful personal narratives, which I actually did find often illustrating).

I have always wondered whether unconscious bias training should be a requirement. Although I have never implemented personally, I have witnessed unconscious biases in some of the discussions I attended.

Communication skills

Communication skills are essential to the functioning of a research team, and it is important that stakeholders can communicate clearly to ensure stakeholder engagement is effective. Crucially, it is important that the research team understand the stakeholders' communication skills and needs from the outset in order to accommodate them. We argue that communication should be ‘enabled’ by the research team to ensure this criterion does not lead to the exclusion of important voices and perspectives.

The research team should be skilled in facilitating communication and themselves should be able to communicate clearly, without ‘jargon’ when required. This would encourage the greatest diversity. Adequate budget, time capacity, and resource allocation (such as capacity for training in communication) are needed to ensure the practicalities of good communication are met. If these resources are not available to ‘enable’ clear communication, then communication skills may unfortunately become an eligibility criterion for a research project. Research teams should therefore be clear with stakeholders about the support they can offer from the beginning of a research project and report this limitation.

*How can this factor be assessed/implemented?*

Communication skills needed depend on the research project and the extent/mode of communication required by stakeholders. We suggest three stages to this process.

Firstly, assess communication skills. If communication will be conducted orally, then a formal or informal conversation may be sufficient. If written communication is needed, stakeholders may need to complete an application form, written task, or pre-engagement survey. Another option is using existing evidence of communication such as examining social media profiles and examining participation in meetings.

Secondly, consider what resources are needed to bring every stakeholder up to a level playing field and decide what resources the team can provide. If stakeholders have specific communication needs, a good starting point is simply asking them how to facilitate their communication.

Thirdly, once you have this information select stakeholders accordingly.

*Quotes from co-authors describing examples of each criterion*

No examples given.

Commitment and time capacity

It is important to ensure that the stakeholder can dedicate the necessary time to complete the project within the timeframe. Stakeholders’ commitment to the research project and their level of dedication will greatly affect the chance of success. Commitment will help stakeholders find the time and resources to complete the task. Research teams could encourage motivation in stakeholders by building rapport, outlining the project’s significance, and benefits for them as individuals and members of the stakeholder group.

Consider what resources are available for stakeholders with limited time availability before commencing stakeholder identification. Consider what can be done to minimise the burden on stakeholders, be aware of time capacity becoming a discriminatory eligibility criterion and try to minimise this. If time capacity is an issue, then bringing in more stakeholders and delegating tasks more broadly may help.

*How can this factor be assessed/implemented?*

Researchers should communicate clearly with the stakeholder what will be required, in terms of time commitment, training, deadlines and meetings etc. and ask the stakeholder whether they can commit to the project requirements and have sufficient time capacity. Consider having a ‘stakeholder contract’. Trust that stakeholders will follow through with their commitment.

*Quotes from co-authors describing examples of each criterion*

This sometimes occurs in publications in general - people might not be able to commit the time to be a full author, but they might be able to contribute parts (and be acknowledged as having done so in the final publication).

I find regular scheduled check-ins are a good motivator on a project with diverse members, to make sure everyone is on track, and also to provide support where it's needed.

Conflicts of interest

It is important to consider the relationship of interest for all stakeholders, whether they may impact input, participation, and research outcomes.

The interests of the stakeholder group that the individual is representing should not be considered a conflict of interest. However, stakeholders may hold conflicts of interest at the individual level (e.g., financial, intellectual), and/or through their institutional affiliation(s). These should be considered carefully as to how they may bias the project.

The research team should have processes for disclosure and management of conflicts of interest. Management could range from simple declaration and publishing disclosures to exclusion of the individual.

*How can this factor be assessed/implemented?*

Conflict of interest assessment is usually self-declared by individual stakeholders often using a conflict-of-interest form.

*Quotes from co-authors describing examples of each criterion*

Personally, I have been engaged in an activity where conflict of interest must be declared, and I haven't come across anyone where I have conflict of interest… Most of the activity I engage in is an obligation associated with my professional practice.

The ICMJE have a useful disclosure of interest form: <http://www.icmje.org/disclosure-of-interest/>

Training, support, and funding needs

Research teams should be clear on the resources (e.g., training, support, and funding) they are able to offer to stakeholders before embarking on stakeholder selection. Research teams should be aware that stakeholder representatives will need varying levels of support throughout engagement. Training and support could be formal or informal and could be outsourced or conducted by the research team. Resources needed to engage stakeholders may include software, reading materials, special equipment, computer, and internet access. Funding should be provided to stakeholders as a compensation for their time and effort, and a recognition of their expertise, especially if they are not financially supported from other sources. In addition, ideally meals, accommodation, transport, and other expenses should be fully reimbursed. Research teams need to make potential stakeholder representatives aware of any training they will be expected to undertake and ask them about any training needs they have.

Ideally neither training, support, nor funding should be a factor in choosing stakeholder representatives; research teams should be ready and able to equip stakeholders with what is needed to engage them. This involves planning, organising a budget, and factoring funding for stakeholder training in grant applications. Some research institutions offer training which reduces the impact on project resources. It may be possible to design the process and research in such a way so that training and support needs are minimal, and you are able to work within stakeholders' existing skill set. For example, consider using software that stakeholders are familiar and comfortable with, arrange meetings at times and durations that suit them.

At times, budgetary constraints may impact training, support, and funding, and consequently the research process and the experience for stakeholders. If funding is not provided, there may be power imbalances with those financially challenged being underrepresented. If any of these becomes an eligibility factor, be transparent in reporting and consider the impact this may have had on the team and research outcomes.

Note: to engage effectively with stakeholders, the research team is likely to need training, support, and members with experience. Ensure there is time and budget for this side of the process as well.

*How can this factor be assessed/implemented?*

Assessment depends on the research and tasks which need to be undertaken. To assess training and support, an informal discussion with the stakeholder may be sufficient. More formal methods include performing a needs assessment, interviews, or looking at an individuals’ CV. Funding needs should be calculated ahead of time by considering the time demands required of stakeholders along with any other stakeholders. Stakeholder time should be paid at the rate commensurate with the hourly rates of at least junior research staff. These training, support, and funding provisions should be offered to stakeholders leaving it to them to decide which to accept and which are not needed.

*Quotes from co-authors describing examples of each criterion*

Training is the teaching and learning activities carried on for the primary purpose of helping members of an organization acquire and apply the knowledge, skills, abilities, and attitudes needed by a particular job and organization. So the training I have acquired over the years has prepared me to carry out the various tasks I have engaged in including this activity.

The NFRF research program places a nice emphasis on early career researchers.

In my prior experience in adapting guidelines, we provided literature to read prior to the meeting and then a short presentation at the beginning of the meeting.

Removed factors

The following factors were removed as most co-authors suggested that research teams do not need to consider these in stakeholder identification. Power sharing is more an issue for the research team to facilitate rather than something to consider when identifying stakeholders. Points of view are hard to quantify and something that changes over time. It is also perhaps over management to consider this in stakeholder identification. Co-authors felt balance of perspectives is important in a researcher-stakeholder team and this will be facilitated using all these criteria, making this specific criterion redundant.

*Power sharing*

It is important to consider stakeholder ability to share power with others as power-sharing is essential to multi-stakeholder work where important perspectives must be balanced. If an individual stakeholder is unable to share power, this can make conversations difficult and break down trust in the team.

The research team has responsibility to manage power imbalances. Communicate expectations early in the process and consider having a power sharing mission statement signed by stakeholders. Some decision-making structures can help overcome dominant individuals. Research team construction may benefit from a lead/chair from the research team and a stakeholder co-chair or deputy to make sure the stakeholder perspective is given equal weight. Having experienced facilitators who expect and prepare for an element of power discrepancy may help mitigate this.

How can this factor be assessed/implemented?

Assessment of power sharing could be conducted with a pre-screen asking about perspectives, values assessment, interview or survey, and examining previous projects the individual has worked on.

Quotes from co-authors describing examples of each criterion

I currently work in some very hierarchical environments and those who are steeped in those environments and used to holding power, leading, and dominating conversations may be hard to involve in teamwork. It may be useful to develop strategies for dealing with those who tend to dominate or disengage.

…in the activity we had in my place of work, I had to get everyone involved by sharing responsibility. It is called decentralization of power. When people are involved by giving them a particular task, they take responsibility for it. They become accountable.

We always have very experienced facilitators moderating groups where we know power imbalances are inevitable.

*Point of view*

Points of view include perspectives of an individual derived from their experience, expertise, or position. These can be shaped by values, work experience, or fact-based judgements and may change over time.

Seeing research through the lenses of different stakeholders can reveal different meaningful perspectives. A range of points of view is useful to ensure project outcomes are representative and avoid bias as much as possible. Having a range of stakeholders will give different points of view, but this could also vary quite widely within one 'type' of stakeholder. It is important to assure stakeholders that all points of view are appreciated.

How can this factor be assessed/implemented?

Ask stakeholders what perspectives they bring during a formal or informal discussion or ask for a short profile about their passions etc.

Quotes from co-authors describing examples of each criterion

Working on projects before, having a variety of points of view added richness to the project.

…in Feb with MUSE, I introduced myself with my country, work status, and photo.
I didn't share other personal information that could be relevant to my views: e.g., family, ancestry/religion …

When organising events, we always include people from different parts of the political spectrum but try to ensure they connect to the mission (ending homelessness) in the same way

*Balance*

As a criterion balance refers to the group of stakeholders as a whole rather than an individual stakeholder. Balance can be applied to many of the other criteria. For example, inclusivity could be applied to achieve the relevant balance, researchers may look for balance in the previous stakeholder engagement of their group, and influence may be something which should be balanced within the stakeholder group. For other criteria, balance may not be desirable. For example, an ideal stakeholder group would contain individuals who are all wholly committed to the research, and who all hold research relevant values.

How can this factor be assessed/implemented?

Balance depends on the purpose of the research. Researchers can achieve balance by getting to know stakeholders well to mitigate imbalances. It is important to recognise imbalances and be aware of how individuals within the stakeholder team will interact with each other to make up the stakeholder group. If imbalance is detected in one area, researchers could consider going beyond their stakeholder group to a larger organisation for consultation on a particular issue.

Quotes from co-authors describing examples of each criterion

No examples given.

References

1. Welch VA, Petkovic J, Jull J, Hartling L, Klassen T, Kristjansson E, et al. Equity and specific populations. Cochrane Handbook for Systematic Reviews of Interventions. 2019:433-49.

2. Östlin P, Schrecker T, Sadana R, Bonnefoy J, Gilson L, Hertzman C, et al. Priorities for research on equity and health: towards an equity-focused health research agenda. PLoS medicine. 2011;8(11):e1001115.

## Appendix 7 – Full description of factors, and important considerations for research teams utilising factors, in the identification and invitation of individuals in a multi-stakeholder research partnership

| **Factors** | **Description** | **Considerations for health research teams** |
| --- | --- | --- |
| Highly desirable | | |
| Ability and willingness to represent stakeholder group | No single person can be expected to represent the views of everyone in a stakeholder group.  A stakeholder may identify as being part of multiple stakeholder groups simultaneously (e.g., policy maker and patient). In this situation it is often not possible to compartmentalise and represent a single group.  If an individual is asked to join the team as a member of a specific stakeholder group, make clear which role(s) they are being asked to fulfil. Consider whether they have the skills to take on this role effectively, such as imagination, empathy, and the ability to look beyond their personal situation. | - If individuals are asked to represent their stakeholder group (keeping in mind they cannot feasibly be expected to represent all perspectives from that group), do they have the skills to take on this role effectively? - Do potential stakeholders belong to more than one stakeholder group (e.g., policy maker and patient) and if so, has it been made clear which group(s) they are being asked to represent (considering that compartmentalising these roles is often not possible)? |
| Commitment and time capacity | A stakeholder’s commitment and dedication to an activity will affect the chance of success. Commitment is fed by motivation which can be encouraged by building rapport, highlighting the projects’ importance, and outlining benefits to the individual and to those in a similar position to them.  Communicate with the stakeholder what will be required (i.e., time commitment, training, deadlines, and meetings etc.) and ask whether the stakeholder can meet project requirements.  Think about what can be done to minimise the commitment burden on stakeholders. Be aware of time capacity becoming a discriminatory eligibility criterion and try to minimise this by being flexible and adapting to stakeholder needs. Consider recruiting more stakeholders and delegating tasks broadly. | - Can stakeholders dedicate the necessary time to the project? - Are stakeholders motivated to take part in the research project and committed to its success? - Have stakeholders been encouraged by building rapport, highlighting the importance of the project and its potential impact? - Have stakeholder requirements (i.e., training, deadlines, meetings) been clearly communicated? Are stakeholders able to meet these requirements? - What can be done to minimize requirements on stakeholders’ time and to adapt to stakeholder needs? |
| Communication skills | Communication skills are essential to the functioning of a research team, and it is important that both stakeholders and researchers can communicate clearly.  If a stakeholder can communicate, in any way, and they meet other important requirements, they should be invited. A stakeholders’ communication skills should be supported by the research team to ensure this factor does not incur the exclusion of important voices and perspectives. Examples include ensuring budget for translators if a stakeholders’ primary language does not match the research team, or using technology to support those with hearing impairments.  Adequate budget, time capacity, and resource allocation ensure practicalities of good communication are met. If these resources are not available to support stakeholders, communication skills may become an eligibility criterion. In this case, it should be made clear to stakeholders and transparently reported in project outputs.  The research team should be skilled in facilitating communication and able to communicate clearly, without jargon when required. | - Can stakeholders communicate clearly and effectively? What can be done to support communication and ensure this does not lead to the exclusion of important voices and perspectives? - If resources to support stakeholders in communication are not available, has this been made clear to stakeholders and transparently reported it in project outputs? - Can research team members communicate clearly with stakeholders, without jargon when required? |
| Financial and non-financial relationships and activities, and conflicts of interest | Consider possible competing relationships and activities and how these influence input, participation, and research outcomes. Stakeholders may hold competing interests at the individual level (e.g., financial, intellectual, or personal), and through institutional affiliation (e.g., financial, or cultural).  The interests of the stakeholder group that the individual is representing should not be considered a competing interest.    It is likely all individuals will have some form of competing financial and/or non-financial relationships and activities. Decide which will be accepted and managed, and which will result in exclusion of the stakeholder. Ensure transparent reporting of all activities and relationships that may be considered competing interests. | - What are potential stakeholders’ conflicts of interest (i.e., financial, intellectual or personal) at the individual and institutional level and how might these impact their participation in the project and research outcomes? - Is there a process for disclosure and management of conflicts of interest? |
| Expertise or experience | Both ‘technical knowledge’ (expertise – which can include e.g., clinical, and methodological) and ‘lived experience’ (experience) have important benefit. Consider what is required for the project and seek to involve stakeholders with expertise and/or experience to create an informed group. It is likely that a balance of expertise and experience will be useful. | - Do stakeholders have relevant technical knowledge (e.g., clinical, and methodological expertise) or lived experience? - Has a balance of both expertise and experience been sought? |
| Inclusivity (equity, diversity, and intersectionality) | Research teams should be committed to actively and openly supporting and promoting inclusivity by considering equity, diversity, and intersectionality when selecting stakeholders.  Think about how stakeholder identification and invitation may influence health inequity e.g., leading to avoidable and unfair differences in health.  Consider the characteristics and demographics of the population that the research aims to serve and ensure the stakeholder group reflects the diversity in this population to ensure results are generalizable and useful to those who need them.  Individuals may be affected by discriminatory social structures in more than one way. Consider how intersectionality (i.e., the intersections among different social hierarchies, such as class and gender, and their cumulative impacts on health inequities (2) influence stakeholder selection and engagement.  Consider how biases within the research team or organisation (e.g., preconceived judgements for or against a particular individual or group) could create advantages or disadvantages to the stakeholders selected and the research output. Consider undertaking training in these issues to be able to effectively address them.  These concepts may have different understandings and interpretations in different countries and communities; it is important to be aware of potential cultural sensitivities relating to this factor. | - How could stakeholder identification influence health inequity i.e., avoidable, and unfair differences in health? - Are traditionally marginalized stakeholders included? - Has there been consideration of the characteristics and demographics of the population that the research aims to serve and are stakeholders selected to reflect this diversity? - How might intersectionality (i.e., the intersections among different social hierarchies, such as class and gender, and their cumulative impacts on health inequities) influence stakeholder identification and engagement? - Does the research teams’ own biases (e.g., preconceived judgements for or against a particular individual or group), or its organizations’ policies or practices disadvantage individuals’ intersecting categories? - Has the research team received training in these issues, or consulted online resources, to enable them to effectively address these?   Note: keep in mind that these concepts may have different understandings and interpretations in different countries and communities; it is important to be aware of potential cultural sensitivities relating to this factor. |
| Training, support, and funding needs | Be clear about the resources (e.g., training, support, and funding) available to stakeholders before embarking on stakeholder identification. Whilst identifying stakeholders, communicate any training stakeholders will be expected to undertake and be aware of the training needs stakeholders possess.  Ideally, neither training, support, nor funding should be an eligibility factor in choosing stakeholders and research teams should be able to equip stakeholders with what is needed to engage. This will involve planning ahead, organising a budget, and insuring grant applications have factored in sufficient funding. This may include training and support for the research team as well.  Budgetary constraints may mean it is not feasible to provide training, support, and funding. If any of these become an eligibility factor, researchers must be transparent in reporting and consider the impact on research outcomes. | - Are you clear about the level of training, support, and funding you are able to offer to stakeholders before embarking on stakeholder identification? - Has every effort been made to equip stakeholders with what is needed to engage? - Have stakeholders been consulted about the training, support and funding needs they have, and how these will be met? - What are the training needs of the research team to help facilitate effective stakeholder engagement? |
| Desirable | | |
| Influence | A stakeholder’s level of influence may positively affect research output, for example by improving the impact of a research project via effective research dissemination. It may also lead to bias (e.g., potentially through having loyalties or pre-conceived notions) or may negatively contribute to power dynamics in the research team.  Consider a stakeholder’s level of influence. Selecting for influence may lead to inequity by excluding people who have not had opportunity to influence the research field. It may be appropriate to involve stakeholders with a range of influence to create a balanced team. | - Do stakeholders have influence within the research field? - What type of influence do they have and how might it affect the work positively and/or negatively? |
| Previous stakeholder engagement | It can take time to train both research teams and stakeholders to work effectively together. Working with the same individuals over time can be a positive sign of a healthy, productive, working relationship.  New stakeholders are also likely to contribute a lot to the research project through different and fresh perspectives. Involving new stakeholders will increase the size and diversity of the pool of experienced stakeholders to engage in research.  It is beneficial for research teams to be aware of previous stakeholder engagement in stakeholder identification, and aim to balance previous experience with new perspectives. | - Do potential stakeholders have previous stakeholder engagement experience? - Have potential advantages and disadvantages of both novice and experienced stakeholders been considered? - If appropriate, have new stakeholders been identified to engage? |
| Research relevant values | A research project may require general research values to be adopted by stakeholders, for example a universal set of values or a set of values by the research team or the organisation they are affiliated with. These values may include traits such as openness, respect, and integrity.  In contrast, if research teams use personal values for stakeholder identification, this could bias selection. | - What research relevant values (not personal values) are essential and/or desirable for the research project? (i.e., a universal set of values or a set of values held by the research team or the organisation they are affiliated with). - What can be done to communicate these values to stakeholders and assess these in potential stakeholders? |

## Appendix 8 - Results from the quantitative and qualitative analysis of the factor categorisation in stage 2

| **Ten factors** | **Quantitative analysis: # of votes** | | | **Qualitative analysis: summary of qualitative comments and executive group discussion** | **Final categorisation** |
| --- | --- | --- | --- | --- | --- |
|  | **Highly desirable** | **Desirable** | **Exclude** |  |  |
| Inclusivity (equity, diversity, and intersectionality) | 14 | 1 | 1 | Comments generally agree this factor is valuable and important. Some comments around the feasibility of finding willing individuals to meet the level of inclusivity ideally required. | Highly desirable |
| Financial and non-financial relationships and activities, and conflicts of interest* | 12 | 1 | 2 | Comments describing complications in what constitutes a conflict of interest but wide agreement that it is important to consider, for the whole research team, activities and relationships that may influence the validity of research when selecting stakeholders. Some comments note that competing activities and relationships are often present and what is more important is how they are managed by the research team. | Highly desirable |
| Commitment and time capacity | 11 | 3 | 2 | Comments acknowledge the importance of this factor being considered in stakeholder selection. Emphasis on the importance of the research team being clear on expectations from the beginning and the stakeholder being transparent about their availability. Comments stress the need for flexibility from researchers, to meet stakeholder needs. | Highly desirable |
| Expertise or experience* | 10 | 3 | 2 | Comments acknowledged it is important for a health research team to consider stakeholder expertise and experience. It was noted this is often the main reason for their involvement. Comments reflected that researchers should consider getting a balance of expertise and experience in the researcher-stakeholder team. | Highly desirable |
| Training, support, and funding needs* | 8 | 7 | 0 | Comments agree it is important for research teams to plan for training, support, and funding needs before stakeholder selection and consider this factor in stakeholder selection, being clear with stakeholders about what can be offered. Comments note that every effort must be made to be inclusive of stakeholders with limited time and resources. | Highly desirable |
| Communication skills* | 8 | 5 | 2 | Comments describe the importance of a stakeholders’ communication skills and the important role of researchers in ensuring effective communication. Comments emphasise that a stakeholders’ ability to convey their perspective is important and that researchers need to support stakeholders with this. | Highly desirable |
| Ability and willingness to represent stakeholder group | 7 | 4 | 5 | Some comments noted the usefulness of ensuring stakeholders are able to fulfil their role of representing their stakeholder group, if this is necessary in a project. However, comments also highlighted two key complexities with this factor. Firstly, caution was expressed that one individual should not be expected to represent the views of everyone in their stakeholder group. Secondly, it should be acknowledged that a stakeholder may identify as being part of multiple stakeholder groups (i.e., policy maker and patient) and in this situation it is unreasonable to ask individuals to compartmentalise and represent a single group. Discussion within the executive group led to agreement that this factor should be included for consideration by health research teams, with the important caveats mentioned. | Highly desirable |
| Research relevant values* | 5 | 3 | 7 | Split consensus. Some commented it is important that everyone in a research team can agree on research values at the beginning of a project, noting people can have divergent opinions but matching research values. It was also commented that it is hard to evaluate people’s research values. Some stated this is academic and not of interest. Executive group discussion highlighted the importance of stakeholders and researchers being able to degree on underpinning research values, e.g., the Declaration of Helsinki. A lack of agreement to key research values could derail the project. The executive group felt research values should be included for consideration by health research teams. | Desirable |
| Influence* | 1 | 11 | 3 | Comments suggested it would be helpful to find out about stakeholders’ influence and be aware of it, though it can be difficult to determine. Stakeholder influence can affect research impact and power dynamics. Co-authors commented that more influence is not always better, and depends on the research area or project. A balance of influence may be desirable. | Desirable |
| Previous stakeholder engagement | 2 | 7 | 6 | High level of ‘exclude’ votes in this factor were mostly accompanied by comments stating that it should not be used to prevent recruitment of novice stakeholders. However, all other comments were in favour of this factor but expressed the importance of including stakeholders both with and without experience of working on research projects before. The aim of this would be to balance previous experience with new perspectives, make sure not to prevent recruitment of new stakeholders, and to give people a chance to develop experience. | Desirable |
| *Note: one respondent did not select from pre-designated groupings for this factor. | | | | | |
